# Factor VII deficiency and developmental abnormalities in a patient with partial monosomy of 13q and trisomy of 16p: case report and review of the literature

**DOI:** 10.1186/1471-2350-7-2

**Published:** 2006-01-13

**Authors:** Brian P Brooks, Jeanne M Meck, Bassem R Haddad, Claude Bendavid, Delphine Blain, Jeffrey A Toretsky

**Affiliations:** 1 National Eye Institute, USA; 2 National Human Genome Research Institute, National Institutes of Health, Department of Health and Human Services, Bethesda, MD; 3 Department of Obstetrics/Gynecology and Oncology, USA; 4 Lombardi Comprehensive Cancer Center; 5 Pediatric Hematology/Oncology, Georgetown University Hospital, Washington, D.C; 6 CNRS UMR 6061 Génétique et Développement, Université de Rennes 1, Groupe Génétique Humaine, IFR140 GFAS, Faculté de médecine, Rennes, France; 7 National Eye Insitute/National Human Genome Research Institute, Building 10, Room 10N226; 10 Center Drive, Bethesda, MD 20892

## Abstract

**Background:**

Unbalanced chromosomal translocations may present with a variety of clinical and laboratory findings and provide insight into the functions of genes on the involved chromosomal segments.

**Case Presentation:**

A 9 year-old boy presented to our clinic with Factor VII deficiency, microcephaly, a seizure disorder, multiple midline abnormalities (agenesis of the corpus callosum, imperforate anus, bilateral optic nerve hypoplasia), developmental delay, hypopigmented macules, short 5^th ^fingers, and sleep apnea due to enlarged tonsils. Cytogenetic and fluorescence *in situ* hybridization analyses revealed an unbalanced translocation involving the segment distal to 16p13 replacing the segment distal to 13q33 [46, XY, der(13)t(13;16)(q33;p13.3)]. Specific BAC-probes were used to confirm the extent of the 13q deletion.

**Conclusion:**

This unique unbalanced chromosomal translocation may provide insights into genes important in midline development and underscores the previously-reported phenotype of Factor VII deficiency in 13q deletions.

## Background

The identification of chromosomal breakpoints in association with variations in human phenotypes often leads to the discovery of novel genes or the characterization of the clinical importance of known genes. Patients who present with a known Mendelian disorder, but who also exhibit developmental delay or other, seemingly unrelated conditions, may harbor a chromosomal abnormality. We present the case of a child who presented with asymptomatic Factor VII deficiency at age 9 who was found to have an unbalanced 13;16 translocation associated with several other developmental abnormalities.

## Case presentation

### Clinical presentation

A 9 year-old male presented with Factor VII deficiency and multiple congenital abnormalities. Prior to her pregnancy with this child, his mother (currently 50 years old) had nine *in vitro* fertilization attempts that ended in miscarriage. One of these miscarriages was documented as trisomy 21 at 18 weeks gestation. This patient was one twin of a fraternal twin pregnancy conceived on the 10^th ^round of in vitro fertilization. His sister was born healthy and her cytogenetic evaluation has not been performed. This twin pregnancy was complicated by premature labor requiring 13 weeks of maternal bed rest prior to delivery via C-section at 36 weeks gestation. The patient's birth weight was 3 lbs, 14.5 oz and his Apgar scores were 9 and 9 at 1 and 5 minutes, respectively.

At birth, the patient was noted to have an imperforate anus that was repaired with a colostomy at 15 days of life. An ultrasound of the brain showed agenesis of the corpus callosum, which was later confirmed on MRI. Both an echocardiogram and a renal ultrasound were normal.

For the first year of life, the patient failed to thrive. He underwent two successful operations for his imperforate anus at ages 9 and 11 months, after which he began to gain weight appropriately. Development was delayed; he sat at 9 months, pulled up at 11 months, walked at 22 months, and spoke single words at 15 months. His pediatricians noted "borderline microcephaly" and low muscle tone. He has been receiving speech therapy and physical therapy since age 1. At age 6 years, he developed *grand mal* seizures which have been successfully managed with medication. He had repeated upper respiratory infections and was noted to have enlarged tonsils that may have contributed to sleep apnea. As part of his pre-operative work-up for tonsillectomy, he was discovered to have elevated prothrombin and partial thromboplastin times of 21.3 seconds and 36.8 seconds, respectively. Factor VII deficiency (17% of normal) was subsequently diagnosed; Factor X levels were 54% of normal. The patient, however, has never had a severe bleeding disorder. It was the combination of Factor VII deficiency and multiple congenital malformations that prompted the genetics work-up, including karyotype. He had abnormal tooth eruption and required dental surgery and frenulectomy. Neither surgery required coagulation assistance.

The patient's parents are healthy, as is his fraternal twin sister and a younger brother, age 7. Review of systems was notable for chronic constipation requiring daily enemas. He is currently on Depakote 325 mg bid and has no known drug allergies.

**Figure 1:**
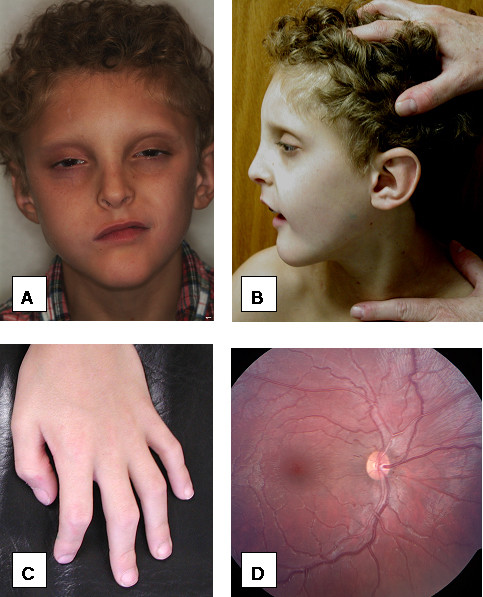
These photographs demonstrate the mild dysmorphic features of the patient, including microcephaly, a triangular and mildly asymmetric face, prominent forehead, slightly downslanting and almond-shaped palpebral fissures, mild ptosis, high nasal bridge and a small chin (panels A and B). He had bilateral 5^th ^finger clinodactyly (panel C) and bilateral optic nerve hypoplasia (panel D).

On physical examination (Figure 1), the patient's height is 51 inches (10^th ^centile), his weight is 42 pounds (3^rd ^centile) and his head circumference is 49.5 cm (<3^rd ^centile, the 50^th ^centile for a 2 year-old). His inner and outer canthal distances are at 97^th ^centile. He has thick, curly hair with one hair whorl and a normal posterior hairline. His forehead is prominent and his face is triangular. His palpebral fissures are somewhat almond-shaped and upslanting. He has mild ptosis. He has a high nasal root, hypoplastic alae nasi with prominent columella, a thin upper lip, downturned corners of the mouth, and a small, pointed chin. He has some mild facial asymmetry. His ears are normally placed and rotated without tags or pits; however, there is a slightly flattened appearance to both auricles. His palate is high arched and he has a small notch in his uvula. He has several dental caps and his lower front teeth are abnormally rotated. His chest is normally shaped without pectus deformity. His chest circumference and inter-nipple distance were 50^th ^and 75^th ^centiles, respectively. He has multiple hypopigmented macules on his torso and one café-au-lait spot on his left shoulder measuring approximately 3 cm × 2 cm. He has approximately 10 degrees of scoliosis, mild lordosis and a small sacral dimple. There are surgical scars from his colostomy and anal repair. He has normal male genitalia, Tanner Stage 1. He has decreased elbow and shoulder extension. His distal extensibility is normal. His 5^th ^fingers are slightly short relative to the 4^th ^distal inter-phalangeal crease and show clinodactyly. His palmar creases are normal. Heart, lung, and abdominal examinations were normal. His knees were normal and he has pes planus.

On ophthalmologic examination, the patients best-corrected visual acuity is 20/50 OU with a +6.25 + 1.25 × 95 OD and +6.25 + 1.75 × 95 OS prescription. He has no measurable stereopsis. Color vision, ocular ductions, ocular alignment, pupillary examination, confrontational visual fields, and slit lamp examination were all normal. Dilated fundoscopic examination showed bilateral, mild optic nerve hypoplasia.

Endocrine studies, including a thyroid panel, a random cortisol, prolactin, and follicle-stimulating were within normal limits. The patient's bone age is at the upper limits of normal by Pyles and Greulich critreria.

### Cytogenetic analysis

**Figure 2:**
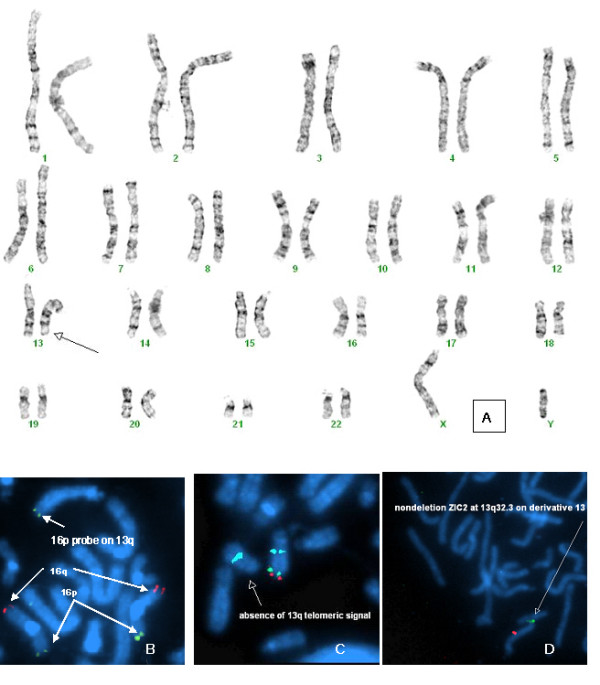
Routine cytogenetics identified a translocation of material of unknown origin onto 13q33 (panel A). Using probes specific for subtelomeric sequences, a translocation was identified from 16p (green) onto 13q (panel B, red probe is for 16q). Similarly, two probes for distal 13q (red and green probes, panel C) were absent from one chromosome 13 identified using an aqua-labeled proximal 13q probe. A FISH BAC probe specific for the *ZIC2* gene on 13q (panel D, green probe) confirms that it is not deleted in this patient.

Routine karyotyping showed a translocation of material of unknown origin onto 13q33 (Figure 2). FISH probes for all telomeres were used to confirm the presence of an unbalanced translocation with distal 16p (16p13.3 → ptelomere) translocated to 13q33. Thus, the patient is monosomic for genes at 13q33 and distal, as well as trisomic for genes at 16p13.3. The nomenclature for this karyotype is 46, XY, der(13)t(13;16)(q33;p13.3). Because the transcription factor gene, *ZIC2*, lies close to the breakpoint and because mutations in *ZIC2* are known to cause holoprosencephaly (a midline defect), we confirmed the breakpoints by performing FISH analysis using *ZIC2* specific bacterial artificial chromosome (BAC) probes, RPCI 11 12-G12 selected from NCBI and Ensembl databases (BACPAC Resources, Children's Hospital Oakland Research Institute, Oakland, CA). These FISH studies confirmed that *ZIC2* was present on both chromosome 13's and that the breakpoint was distal to 13q32, consistent with our impression that the breakpoint is at 13q33. Cytogenetic analysis of both parents was recommended given the history of previous miscarriages, but has not yet been pursued by the family.

## Conclusion

**Figure 3:**
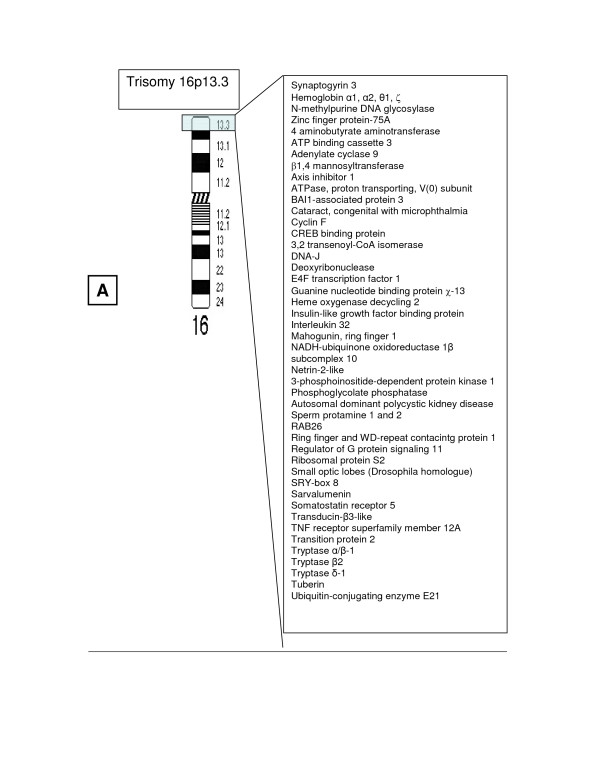
Ideogram of chromosomes 16 with the trisomic region highlighted. Examples of genes known to map to these chromosomal segments are noted, according to the information in build 35.1 of the *Homo sapiens* sequence on the NCBI Map Viewer and Online Mendelian Inheritance in Man. Hypothetical proteins and open reading frames are omitted from the list. In some cases, the exact order of transcripts is ambiguous.

**Figure 4:**
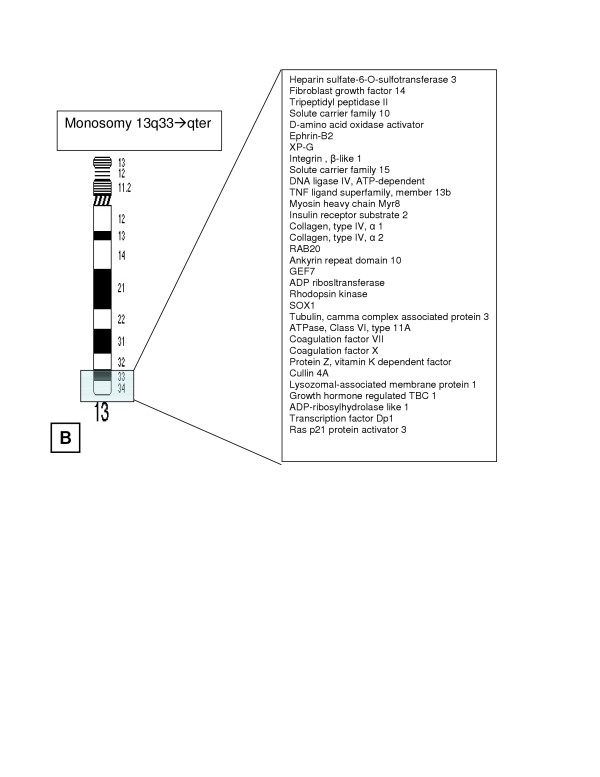
Ideogram of chromosome 13 with the monosomic region highlighted. Examples of genes known to map to these chromosomal segments are noted, according to the information in build 35.1 of the *Homo sapiens* sequence on the NCBI Map Viewer and Online Mendelian Inheritance in Man. Hypothetical proteins and open reading frames are omitted from the list. In some cases, the exact order of transcripts is ambiguous

We report on the clinical and cytogenetics findings in a male with a novel, unbalanced 13;16 chromosomal translocation that resulted in monosomy for genes from 13q33 to the q terminus and trisomy for 16p13.3 genes, the most distal band on the short arm of chromosome 16. Examples of genes that map to these segments are given in Figure 3 and 4. Because chromosome 16 may include imprinted loci, it is possible that the phenotype of this patient could be affected by parent-of-origin effects and/or uniparental disomy for 16p13.3. The presence of several midline abnormalities (e.g., absent corpus callosum, optic nerve hypoplasia, small cleft in the uvula, and anal atresia) suggest a midline field abnormality around the time of gastrulation.[1]

While 13q deletion syndromes are well-recognized,[2] we present a novel translocation associated with 13q deletion and a complex clinical phenotype. The variability in reported phenotypes depends, in part, on the portions of 13q that are deleted and whether other chromosomal abnormalities are involved. Table 1 summarizes published reports of very distal 13q deletions similar to ours. Findings in our patient consistent with 13q deletion include growth and psychomotor retardation, microcephaly, deficiency of coagulation factors VII and X, and mild facial dysmorphisms and asymmetry. His sacral dimple is a finding consistent with the proposal of Luo et al that this region may include genes important in neural tube closure.[3] Similarly, his imperforate anus, muscular hypotonia, microcephaly and dysmorphic features are consistent with the three patients described by Kuhnle et al with sub-telomeric deletions of 13q, although this case is complicated by mosaicism for monosomy 13 and is therefore not included in table 1.[4] Stoll and Alembik describe a patient with 13q33.3→qter with only mild mental retardation, microcephaly, growth delay, a small chin, hypotonia, and a broad forehead[5] and Rivera et al. review six cases with many similar findings, despite a larger 13q deletion.[6] Submicroscopic deletions and/or rearrangements of 13qter have also been reported to lead to "idiopathic" mental retardation.[7]

**Table 1:** Summary of cases with relatively isolated monosomy 13q33-qter.

Chromosomal Abnormality	Clinical Features	Reference
46, XY, del(13)(q33.3)	IUGR; microcephaly; SS; hypotonia; large, low-set ears; hypertelorism; small chin; high/ broad forehead; SPC; mild psychomotor delay	[5]
46, XY, del(13)(q33.2)	IUGR; microcephaly; SS; psychomotor delay; reduced factors VII and X; MR; SS; high nasal bridge; large ears; stiff thumbs; language delay	[35]
46, XY, del(13)(q33.2)	IUGR; microcephaly; SS; speech delay; tapering fingers; transverse palmar crease; high nasal bridge; mild anal prolapse; reduced factors VII and X	[35]
46, XY, del (13)(q33)	Growth and psychomotor retardation; microcephaly; brachycephaly; facial asymmetry; ear anomalies; hypospadias	[36]
46, XX, del(13)(q33)	Psychomotor retardation; hypertelorism; upslanting PF; ear anomalies	[37]
46, XY, del(13)(q33.2)	Lumbosacral myelomeningocele; bilateral cryptorchidism; ambiguous genitalia; microcephaly; telecanthus; short PF; large ears; broad nasal bridge; short philtrum; enamel defects; short neck;	[3]
46, XX, del(13)(q33)	IUGR; growth and psychomotor retardation; microcephaly; hypertelorism; high nasal bridge; ear anomalies	[38]
46, XY, del(13)(q32.3q33.2)	Hirschspring disease; psychomotor retardation; ear anomalies	[39]

Abbreviations: IUGR = intrauterine growth retardation; MR = mental retardation; PF = palpebral fissures; SS = short stature.

Less has been reported on trisomy 16p, perhaps because it is less susceptible than other chromosomal regions to translocation[8] and because complete trisomy 16 – the most frequent autosomal anomaly found in miscarriages – is frequently lethal.[9] Again, there is significant phenotypic heterogeneity reported, in part because most patients described have unbalanced translocations and are trisomic for variable portions of 16p. [10-19] Findings consistent with this anomaly in our patient include microcephaly, a broad nasal bridge, developmental delay, and seizures.[16,20] Cases involving only trisomy of 16p13 are summarized in Table 2. An insertion of 16p13.1→p13.3 into chromosome 1 resulted in mental retardation, short stature, microcephaly, seizures, and multiple dysmorphic features.[21] A 14 year-old male with 46, XY, dup(16)(p13.1→pter) karyotype presented with autism, Tourette's syndrome, short stature, a prominent chin, elongated face, hi-arched palate, small penis/scrotum, poor fine and gross motor movements, and slow basal activity on EEG.[22] Hunter et al. describe a newborn with a 7;16 unbalanced translocation resulting in trisomy of 16p13.1→pter and monosomy of 7p22→pter, chondrodysplasia punctata, absence of the gallbladder, and microcornea.[23] Tschernigg et al. describe a child with "pure" 16pter→p13 due to a tandem duplication of this region, cleft lip/palate, cardiac defects, and club hands/feet. [24]

**Table 2:** Summary of cases with relatively isolated trisomy 16p13.

Chromosomal Abnormality	Clinical Features	Reference
46, XY, der(1), ins(1;16)(q24;p13.1p13.3)pat	MR, SS, microcephaly, mildly dysmorphic faces, proximally inserted thumbs, flexion contractures of PIP joints, deep-set nails, genu valga	[21]
46, XY, dup(16)(p13.1→pter)	autism, Tourette's syndrome, SS, prominent chin, elongated face, hi-arched palate, small penis/scrotum, poor fine and gross motor movements; slow basal activity on EEG	[22]
46, XY, add(16)(p16.3)	microcephaly; short neck, sparse har, hypertelorism, narrow PF, low set ears; bilateral CL/P; club feet/hands; tracheomalacia; VSD, ASD; hypoplastic aorta	[24]

Abbreviations: ASD = atrial-septal defect; CL/P = cleft lip/palate; EEG = electroencephalogram; MR = mental retardation; PF = palpebral fissures; PIP = proximal interphalangeal; SS = short stature; VSD = ventricular-septal defect.

Factor VII is a vitamin-K dependent clotting factor in the extrinsic pathway that – when deficient – is inherited as an autosomal recessive trait and produces an elevated partial thromboplastin time (PTT) in the face of a normal activated prothrombin time (PT); hemarthrosis; intracranial hemorrhage; hematuria; spontaneous epistaxis and bruising; and genitourinary and gastrointestinal bleeding. Although levels of activity correlate imprecisely with symptoms, it is likely that levels below 2% of normal are required before major symptoms develop. The Factor VII gene maps to 13q34,[25] is alternatively spliced, and has multiple poly-adenylation signals.[26] Pfeiffer et al. describe two cases of sub-clinical factor VII deficiency associated with a 46, XY, t(13;Y)(q11;q34) translocation and probable deletion of a terminal segment of 13q that manifested as elevated PTT.[27] Hewson and Carter described severe Factor VII deficiency in a case of 13q deletion syndrome;[28] while Fukushima et al. found approximately 50% factor V activity in two of three patients with terminal 13q deletions and normal levels in a patient with trisomy 13.[29] Our patient would be predicted to be monosomic for 13q34. The fact that he exhibits less than 50% activity may be related to a polymorphism on his remaining allele causing slightly reduced activity or reduced levels of protein expression. Factor VII activity was not assayed in his parents.

Bilateral optic nerve hypoplasia is sometimes accompanied by midline anomalies of the central nervous system, such as absent corpus callosum, absent septum pellicidum, and pituitary insufficiency.[30] None of the three genes known to produce this phenotype in animal models – namely *netrin* (17p13-p12), *Hesx1* (3p21),[31] and *DCC* (18q21.3)[32] – map to either chromosome 13 or 16, suggesting an uncharacterized regulatory gene for midline CNS development in the abnormal regions. Of note, *netrin-2 -like* gene is located on 16p13.[33] Several other zinc-finger containing proteins of unknown function also map to this region. A three-generation family with dominant congenital cataract and microphthalmia co-segregates with a t(2;16)(p22.3;p13.3) translocation in four balanced carriers and three with monosomy of 16p13.3, suggesting that an important eye developmental gene resides at or near this breakpoint.[34]

## List of abbreviations

ASD = atrial-septal defect; BAC = bacterial artificial chromosome; CL/P = cleft lip/palate; EEG = electroencephalogram; FISH = fluorescent in situ hybridization; IUGR = intrauterine growth retardation; MR = mental retardation; MR = mental retardation; OD = right eye; OS = left eye; OU = both eyes; PF = palpebral fissures; PIP = proximal interphalangeal; PT = activated prothrombin time; PTT = partial thromboplastin time; SHH = sonic hedgehog; SPC = single palmar crease; SS = short stature; VSD = ventricular-septal defect.

## Competing interests

The author(s) declare that they have no competing interests.

## Authors' contributions

JAT initially saw the patient and requested a karyotype. BPB and JMM performed the initial karyotype and the FISH studies to delineate the translocation. BRH and CB performed the FISH using specific BAC probes for ZIC2. BPB and DB performed a complete physical examination, clinical genetics evaluation, and ophthalmologic examination.

## Pre-publication history

The pre-publication history for this paper can be accessed here:


